# HEALTH-RELATED ANXIETY, DOCTOR–PATIENT RELATIONSHIP, AND COMMUNICATIONS IN LATE LIFE

**DOI:** 10.1093/geroni/igad104.2149

**Published:** 2023-12-21

**Authors:** Poshan Dahal, Eva Kahana, Nan Zhou, Yuanchang Zhao, Dyanna Burnham

**Affiliations:** Case Western Reserve University, Cleveland, Ohio, United States; Case Western Reserve University, Cleveland, Ohio, United States; Case Western Reserve University, Cleveland, Ohio, United States; Case Western Reserve University, Cleveland, Ohio, United States; Case Western Reserve University, Cleveland, Ohio, United States

## Abstract

The relationship and communication between doctor and patient have important implications for the quality of health care received in old age. However, their role in health-related anxiety among older patients has received limited attention. In this paper, we explored the association between doctor-patient communication and doctor-patient relationship with health-related anxiety among 661 independent, retirement community-dwelling older adults. The mean age of the respondents was 77.81 years, and the majority (60.54 percent) were women. Our findings show a negative association between health–related anxiety and the doctor-patient relationship (b=-11, p<.05). We also found a negative association between satisfaction with communication and health-related anxiety (b=-.09, p<.05). The relationship between health-related anxiety and doctor-patient relationship and doctor-patient communication remains even after controlling for covariates. Older adults who reported better communication and relationship with their physician had lower health-related anxiety. Compared to Blacks, Whites had significantly lower health-related anxiety (b=-.80, p<.05). For every additional chronic condition, health-related anxiety increased by 0.22 (p< 05). Gender, education, and age, however, were not associated with health anxiety. Overall, our findings support the hypothesis that better doctor-patient relationships and communication are associated with lower health-related anxiety among community-dwelling older adults.

